# Development and Internal Validation of the MELD–ERT Score for Mortality Risk Stratification After Liver Transplantation

**DOI:** 10.3390/jcm15207903

**Published:** 2026-10-13

**Authors:** Bartu Çetin, Murat Zeytunlu

**Affiliations:** Department of General Surgery, Medical Point Hospital, İzmir University of Economics, İzmir 35575, Türkiye

**Keywords:** erythrocyte transfusion, liver transplantation, MELD score, mortality, prognosis, risk stratification, survival

## Abstract

**Background/Objectives:** The Model for End-Stage Liver Disease (MELD) score reflects pretransplant disease severity, but does not capture intraoperative factors that may influence post-transplant outcomes. This study aimed to develop and internally validate a composite MELD–erythrocyte transfusion (ERT) score for mortality risk stratification after liver transplantation (LT). **Methods:** This retrospective, single-center cohort study included 125 adult LT recipients between August 2024 and July 2026. A MELD–ERT score (0–2) was constructed using ROC-derived cut-offs for MELD and intraoperative ERT. Prognostic performance for mortality and overall survival (OS) was evaluated using regression, conventional and time-dependent ROC and survival analyses, with bootstrap internal validation. **Results:** During a median follow-up of 544 days, 30 patients (24.0%) died. MELD and ERT were independently associated with mortality. Mortality increased stepwise across MELD–ERT scores of 0, 1, and 2 (0%, 36.2%, and 59.1%, respectively). The MELD–ERT score showed good discrimination (AUC 0.836, 95% CI 0.776–0.897), with an optimism-corrected AUC of 0.818 after bootstrap validation. Its discrimination was comparable to MELD alone (*p* = 0.660) and superior to ERT alone (*p* = 0.002). Time-dependent AUCs for MELD–ERT were 0.893, 0.930, 0.837, and 0.812 at 3, 6, 12, and 24 months, respectively. OS differed significantly across score categories (log-rank *p* < 0.001), and high-risk patients had a 9.7-month shorter 24-month RMST (95% CI 5.4–14.3; *p* < 0.001). **Conclusions:** The MELD–ERT score provides a simple approach to post-LT mortality risk stratification and demonstrated good discrimination after internal validation. However, it did not significantly outperform MELD alone. External validation is warranted.

## 1. Introduction

Liver transplantation (LT) remains the definitive treatment for selected patients with end-stage liver disease and provides substantial survival benefit across a broad spectrum of disease severity. Accurate assessment of recipient risk is essential not only for organ allocation but also for prognostic evaluation before and after transplantation. The Model for End-Stage Liver Disease (MELD) score has become a cornerstone of LT assessment because of its ability to objectively quantify disease severity and predict short-term mortality. More recently, MELD 3.0 was developed to improve mortality prediction and address limitations of previous MELD-based models, demonstrating improved discrimination compared with MELD-Na [1].

Although MELD was primarily developed to predict wait-list mortality, higher MELD scores have also been associated with adverse outcomes after transplantation. Previous studies have demonstrated an association between greater pretransplant disease severity and poorer post-transplant survival, supporting the prognostic relevance of MELD beyond organ allocation alone [2,3]. Nevertheless, MELD primarily reflects the recipient’s preoperative physiological condition and does not incorporate intraoperative events that may substantially influence postoperative outcomes.

Among perioperative factors, intraoperative erythrocyte transfusion (ERT) has been associated with adverse outcomes following LT. Higher intraoperative transfusion requirements have been associated with reduced short- and long-term survival after transplantation [4]. Furthermore, systematic evaluation of transfusion management in LT has demonstrated an association between perioperative ERT and increased mortality, emphasizing the potential prognostic relevance of transfusion burden during transplantation [5].

Importantly, intraoperative ERT may provide prognostic information that is not fully represented by preoperative disease severity. ERT has been identified as an independent predictor of post-transplant survival, with evidence suggesting that its association with mortality may vary over the postoperative period [6]. Taken together, these findings suggest that pretransplant disease severity and intraoperative transfusion burden may represent complementary dimensions of perioperative risk. However, a simple and clinically applicable prognostic approach integrating these two readily available parameters remains insufficiently explored.

Therefore, we hypothesized that integrating the MELD score with intraoperative ERT requirement could provide a simple and clinically interpretable approach to post-transplant risk stratification. The present study aimed to develop and internally validate a composite MELD–ERT score and to evaluate its association with all-cause mortality and overall survival after LT.

## 2. Materials and Methods

### 2.1. Study Design, Population, and Data Collection

This retrospective, single-center observational cohort study was conducted at İzmir University of Economics Medical Point Hospital, İzmir, Türkiye, and included consecutive adult patients (≥18 years) who underwent LT between August 2024 and July 2026. Patients with insufficient data to determine the MELD score, intraoperative ERT requirement, or survival status were excluded. Both living-donor and deceased-donor LT were included, as were re-transplantation cases.

Demographic, clinical, donor-related, intraoperative, transfusion, and outcome data were retrospectively obtained from electronic medical records, transplant records, operative reports, anesthesia records, and laboratory databases. Recipient variables included age, sex, BMI, BSA, diabetes mellitus, liver disease etiology, MELD score, and re-transplantation status. Donor characteristics included donor type, age, sex, BMI, and recipient-to-donor BSA ratio. Intraoperative variables included warm and cold ischemia times, estimated blood loss, cell saver returned blood volume, and total intraoperative ERT units.

A total of 125 adult liver transplant recipients constituted the final study cohort. All transplantation, perioperative, and transfusion decisions were made according to routine clinical practice and were not influenced by the study.

### 2.2. MELD Score and Intraoperative ERT

Pretransplant disease severity was assessed using the original MELD score, based on serum bilirubin, international normalized ratio (INR), and serum creatinine, using the most recent values available before LT. Intraoperative ERT was defined as the total number of packed red blood cell units administered during the transplantation procedure, as recorded in anesthesia and transfusion records.

### 2.3. Development of the MELD–ERT Score

The MELD–ERT score was developed as a simple additive prognostic score combining pretransplant disease severity (MELD) and intraoperative transfusion burden (ERT). MELD and ERT were selected a priori as complementary components representing preoperative and intraoperative risk, respectively. Both variables were initially analyzed as continuous measures, and their independent associations with all-cause mortality were assessed using a multivariable logistic regression model. Optimal cut-off values were subsequently determined by ROC analysis and used to construct the composite MELD–ERT score.

### 2.4. Determination of Cut-Off Values and Construction of the MELD–ERT Score

ROC curve analyses were performed separately for MELD and intraoperative ERT, using all-cause mortality as the outcome. Optimal cut-off values were determined by maximizing the Youden index (sensitivity + specificity − 1). The identified thresholds were MELD ≥ 22 and ERT ≥ 4 units, which were subsequently used to construct the MELD–ERT score.

Each adverse component was assigned one point: MELD ≥ 22 = 1 point and ERT ≥ 4 units = 1 point; values below these thresholds were assigned 0 points. The MELD–ERT score was calculated as the sum of these two components and ranged from 0 to 2:

- Score 0: MELD < 22 and ERT < 4 units.
- Score 1: either MELD ≥ 22 or ERT ≥ 4 units, but not both.
- Score 2: MELD ≥ 22 and ERT ≥ 4 units.

For exploratory analyses, the score-1 category was further subdivided according to the component responsible for the intermediate-risk classification: score 1A was defined as MELD ≥ 22 with ERT < 4 units, whereas score 1B was defined as MELD < 22 with ERT ≥4 units. These subcategories were used only to explore potential differences in mortality and survival within the score-1 group and did not alter the original 0–2 MELD–ERT scoring system.

Equal weighting was used to maintain a simple and clinically interpretable additive score. The three-level score was used to assess the stepwise association with mortality and overall survival. For binary risk-group analyses, scores 0–1 were classified as low risk, whereas score 2 was classified as high risk, representing the simultaneous presence of both adverse components.

### 2.5. Study Outcome and Follow-Up

The primary outcome was all-cause mortality after LT. Overall survival (OS) was defined as the time from transplantation to death from any cause. Patients alive on 10 August 2026 were censored on that date.

### 2.6. Statistical Analysis

Statistical analyses were performed using IBM SPSS Statistics for Windows, version 26.0 (IBM Corp., Armonk, NY, USA) and R version 4.6.0 (R Foundation for Statistical Computing, Vienna, Austria) within the RStudio environment (Version 2025.05.1+513; Posit Software, PBC, Boston, MA, USA). Normality was assessed using the Shapiro–Wilk test and visual inspection of histograms and Q–Q plots. Continuous variables were presented as median (IQR) and compared using the Mann–Whitney U test, while categorical variables were presented as *n* (%) and compared using the chi-square or Fisher’s exact test, as appropriate.

Associations with all-cause mortality were evaluated using univariable and multivariable logistic regression analyses and reported as odds ratios (ORs) with 95% confidence intervals (CIs). A multiplicative interaction term between continuous MELD and ERT (MELD × ERT) was additionally tested in the logistic regression model to assess whether the association of ERT with mortality varied according to MELD severity. The association between intraoperative ERT and estimated blood loss (EBL) was assessed using Spearman’s rank correlation coefficient. Multicollinearity among MELD, ERT, and EBL was evaluated using variance inflation factors (VIFs). Sensitivity analyses were performed by additionally adjusting the multivariable model including MELD and ERT for estimated blood loss and donor type (living vs. deceased donor) in separate models to assess potential confounding while limiting model complexity given the number of mortality events. Given the imbalance in donor type between the MELD–ERT risk groups, an additional sensitivity analysis was restricted to living donor liver transplantation (LDLT) recipients. The MELD ≥ 22 and ERT ≥ 4-unit thresholds derived in the overall cohort were retained without re-estimation in this subgroup. ROC analysis was used to determine optimal cut-off values using the Youden index and to assess the discriminative performance of MELD, ERT, and MELD–ERT; AUCs were compared using the DeLong test. To account for censoring and variation in follow-up duration, time-dependent ROC analyses were additionally performed using the inverse probability of censoring weighting (IPCW) cumulative/dynamic approach at 3, 6, 12, and 24 months. Time-dependent AUCs were estimated separately for MELD, ERT, and the MELD–ERT score, with 95% confidence intervals obtained using 2000 bootstrap resamples. Internal validation of the MELD–ERT score was performed using 5000 bootstrap resamples. The entire score-development procedure was repeated within each bootstrap sample to estimate optimism in model discrimination, and an optimism-corrected AUC was calculated. Model calibration was additionally assessed using the Brier score and calibration slope, with bootstrap resampling used to evaluate the stability of calibration. Decision-curve analysis was performed across clinically relevant threshold probabilities to explore the potential net benefit of the MELD–ERT score compared with MELD alone. In addition, a model retaining MELD and ERT as continuous variables was evaluated to examine the potential information loss associated with dichotomization. Because follow-up duration varied among recipients, time-to-event analyses were used to evaluate survival outcomes, whereas logistic regression analyses of all-cause mortality were considered supportive analyses and did not account for differences in follow-up duration. OS was estimated using the Kaplan–Meier method and compared using the log-rank test, with patients alive on 10 August 2026 censored on that date. Median follow-up duration was estimated using the reverse Kaplan–Meier method.

Cox proportional hazards regression was used to assess the association between high MELD–ERT score (2 vs. 0–1) and OS, with multivariable adjustment for age and sex, and results were reported as hazard ratios (HRs) with 95% CIs. The proportional hazards assumption was assessed using Schoenfeld residuals. Because the proportional hazards assumption was not satisfied for the MELD–ERT risk group, a restricted mean survival time (RMST) analysis over a 24-month time horizon was additionally performed. All tests were two-sided, and *p* < 0.05 was considered statistically significant.

### 2.7. Ethical Considerations

The study was conducted in accordance with the Declaration of Helsinki and approved by the İzmir University of Economics Health Sciences Research Ethics Committee (approval no. E-97429853-050.04-132084; date: 25 August 2026). Due to the retrospective design, the requirement for informed consent was waived. All patient data were anonymized before analysis.

## 3. Results

### 3.1. Baseline Characteristics

A total of 125 adult liver transplant recipients were included, of whom 103 (82.4%) were classified as low risk (MELD–ERT score 0–1) and 22 (17.6%) as high risk (score 2). The median follow-up duration, estimated using the reverse Kaplan–Meier method, was 544 days (approximately 17.9 months). Recipient age, sex, BMI, BSA, diabetes mellitus, liver disease etiology, and re-transplantation rates were comparable between the groups. Compared with the low-risk group, high-risk patients more frequently underwent living donor transplantation (95.5% vs. 60.2%, *p* = 0.001), had younger donors (33 [26.2–36] vs. 42 [30.5–50.5] years, *p* = 0.001), shorter cold ischemia times (66.5 [48.2–75.2] vs. 74 [63–380] min, *p* = 0.007), and greater estimated blood loss (3000 [2125–3875] vs. 1200 [700–1900] mL, *p* < 0.001). Overall mortality was significantly higher in the high-risk group than in the low-risk group (59.1% vs. 16.5%, *p* < 0.001) (Table 1). Preoperative hematologic and coagulation parameters were additionally evaluated to assess whether baseline abnormalities might have influenced intraoperative transfusion requirements. Preoperative hemoglobin, platelet count, INR, and fibrinogen levels did not differ significantly between the low- and high-risk MELD–ERT groups (all *p* > 0.05; Table 1). In correlation analyses, none of these preoperative parameters showed a statistically significant association with intraoperative ERT requirement.

### 3.2. Logistic Regression Analysis

Univariable logistic regression analysis demonstrated that both the MELD score and intraoperative ERT were significantly associated with overall mortality after LT. Each one-point increase in the MELD score increased the odds of mortality by 19% (OR 1.19, 95% CI 1.10–1.28, *p* < 0.001), while each additional unit of ERT was associated with a 26% increase in the odds of mortality (OR 1.26, 95% CI 1.07–1.49, *p* = 0.006). Body mass index, donor age, and living donor transplantation showed trends toward an association with mortality but did not reach statistical significance. In the multivariable analysis, both the MELD score (OR 1.18, 95% CI 1.10–1.28, *p* < 0.001) and intraoperative ERT (OR 1.22, 95% CI 1.02–1.45, *p* = 0.029) remained significantly associated with overall mortality when included together in the model (Table 2). No statistically significant interaction was observed between MELD and ERT (interaction OR 0.984, 95% CI 0.955–1.014; *p* = 0.288), providing no evidence of a multiplicative interaction between the two variables. Intraoperative ERT showed a moderate positive correlation with EBL (Spearman’s ρ = 0.426, *p* < 0.001). Despite this association, there was no evidence of substantial multicollinearity among MELD, ERT, and EBL, with VIF values of 1.04, 1.71, and 1.70, respectively. In sensitivity analyses, the association between intraoperative ERT and mortality remained significant after additional adjustment for estimated blood loss (OR 1.37, 95% CI 1.04–1.80; *p* = 0.025) and, in a separate model, for donor type (OR 1.21, 95% CI 1.01–1.45; *p* = 0.037). Neither estimated blood loss nor donor type was significantly associated with mortality in these models.

Given the imbalance in donor type between the risk groups, an additional sensitivity analysis was restricted to the 83 LDLT recipients. Using the same MELD ≥22 and ERT ≥4-unit thresholds derived in the overall cohort, mortality increased progressively across MELD–ERT scores, from 0% (0/32) for score 0 to 40.0% (12/30) for score 1 and 57.1% (12/21) for score 2 (*p* < 0.001). The MELD–ERT score retained good discrimination in the LDLT-only cohort (AUC 0.809). When analyzed as an ordinal variable, each one-point increase in the MELD–ERT score was associated with higher odds of mortality (OR 5.48, 95% CI 2.45–12.28; *p* < 0.001). Similarly, patients with a score of 2 had higher odds of mortality than those with scores of 0–1 (OR 5.56, 95% CI 1.91–16.19; *p* = 0.002) (Table 3).

Data are presented as odds ratios (ORs) with 95% confidence intervals (CIs). The multivariable model included MELD score and ERT, which were prespecified as the two components of the MELD–ERT score.

### 3.3. Mortality Across MELD–ERT Score Categories

Based on the identified cut-off values, a simple MELD–ERT score was developed by assigning one point each for a MELD score ≥22 and intraoperative ERT ≥4 units, resulting in a three-level risk score ranging from 0 to 2. Overall mortality increased progressively across the MELD–ERT score categories, from 0.0% in patients with a score of 0 to 36.2% in those with a score of 1 and 59.1% in those with a score of 2 (Table 4).

Within the score-1 category, 37 patients (78.7%) were classified as score 1A (MELD ≥22 and ERT <4 units), of whom 15 died (40.5%), whereas 10 patients (21.3%) were classified as score 1B (MELD <22 and ERT ≥4 units), of whom 2 died (20.0%). Although mortality was numerically higher in score 1A than in score 1B, OS did not differ significantly between the two subgroups (log-rank *p* = 0.221).

### 3.4. Receiver Operating Characteristic (ROC) Analysis

**Figure 1:**
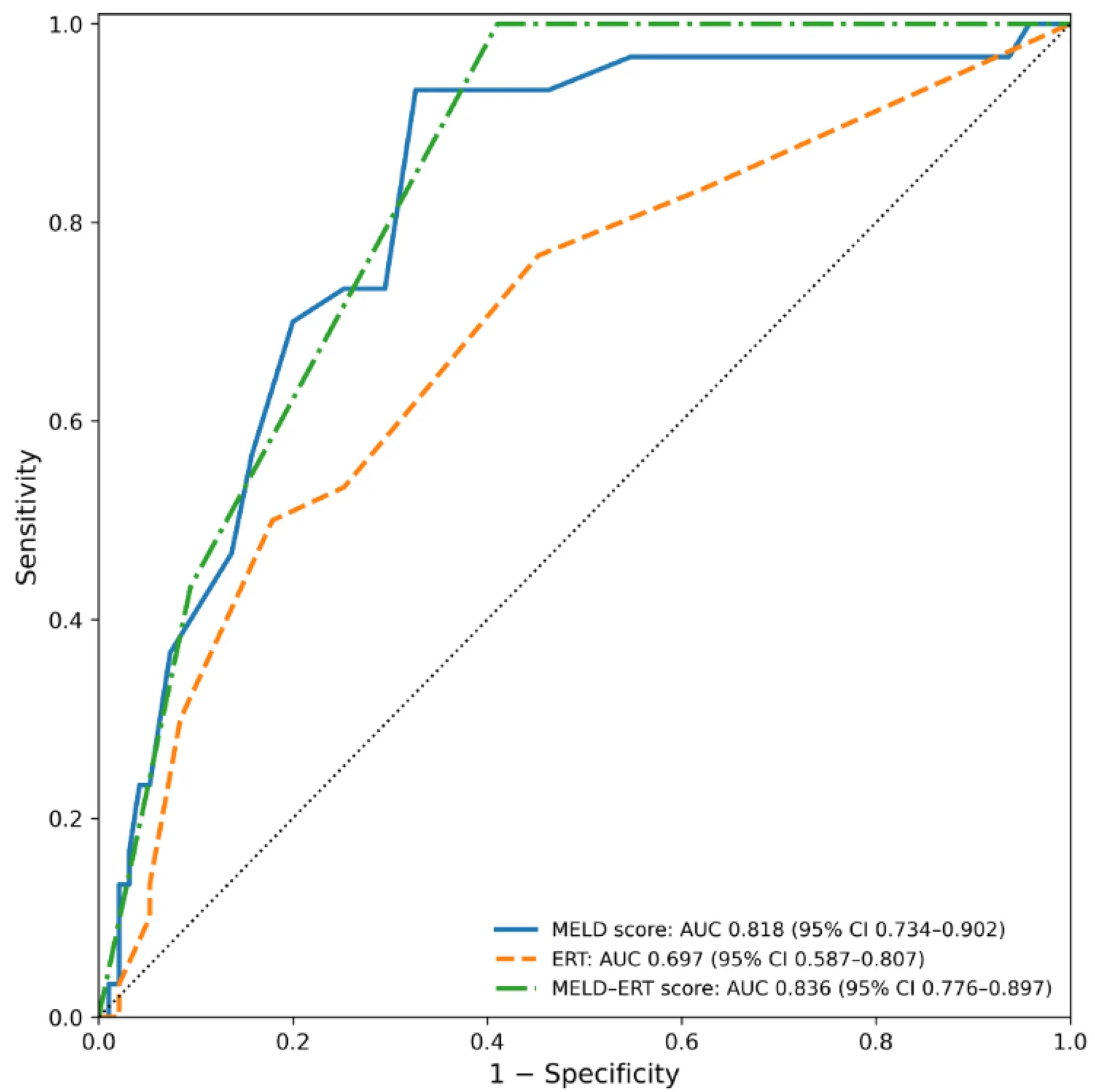
ROC curves of the MELD score, ERT, and the MELD–ERT score for predicting overall mortality after liver transplantation.

ROC analysis identified MELD ≥ 22 as the optimal cut-off for predicting overall mortality, with a sensitivity of 93.3% and a specificity of 67.4%, whereas the optimal cut-off for ERT was ≥4 units, with a sensitivity of 50.0% and a specificity of 82.1%. The MELD–ERT score achieved the highest discriminative performance, with an AUC of 0.836 (95% CI, 0.776–0.897), compared with the MELD score (AUC 0.818, 95% CI, 0.734–0.902) and ERT (AUC 0.697, 95% CI, 0.587–0.807) (Figure 1). Pairwise comparison using DeLong’s test showed no significant difference between the MELD–ERT score and MELD (*p* = 0.660), whereas the MELD–ERT score showed significantly greater discrimination than ERT alone (*p* = 0.002). Internal validation using 5000 bootstrap resamples yielded an optimism-corrected AUC of 0.818, compared with the apparent AUC of 0.836, indicating limited optimism in the discriminative performance of the MELD–ERT score. Additional model-performance analyses showed a Brier score of 0.137 for the MELD–ERT score compared with 0.144 for MELD alone. The bootstrap calibration slope for MELD–ERT was 0.99, indicating good calibration with respect to the calibration slope within the development cohort. Decision-curve analysis showed modest differences in net benefit between MELD–ERT and MELD across clinically relevant threshold probabilities, without a consistent advantage of MELD–ERT over MELD alone. A model retaining MELD and ERT as continuous variables yielded an AUC of 0.837, which was similar to that of the simplified MELD–ERT score (AUC 0.836).

Time-dependent ROC analysis further demonstrated that the MELD–ERT score maintained good discrimination across follow-up. The time-dependent AUCs for MELD–ERT were 0.893 (95% CI 0.793–0.953) at 3 months, 0.930 (95% CI 0.869–0.973) at 6 months, 0.837 (95% CI 0.761–0.905) at 12 months, and 0.812 (95% CI 0.702–0.903) at 24 months. The corresponding AUCs for MELD were 0.857, 0.819, 0.789, and 0.829, respectively, whereas those for ERT were 0.841, 0.875, 0.722, and 0.537. These findings indicate that the discriminative performance of the MELD–ERT score remained relatively stable over time, without demonstrating consistent superiority over MELD alone at every time point (Table 5).

### 3.5. Kaplan–Meier Survival Analysis

**Figure 2:**
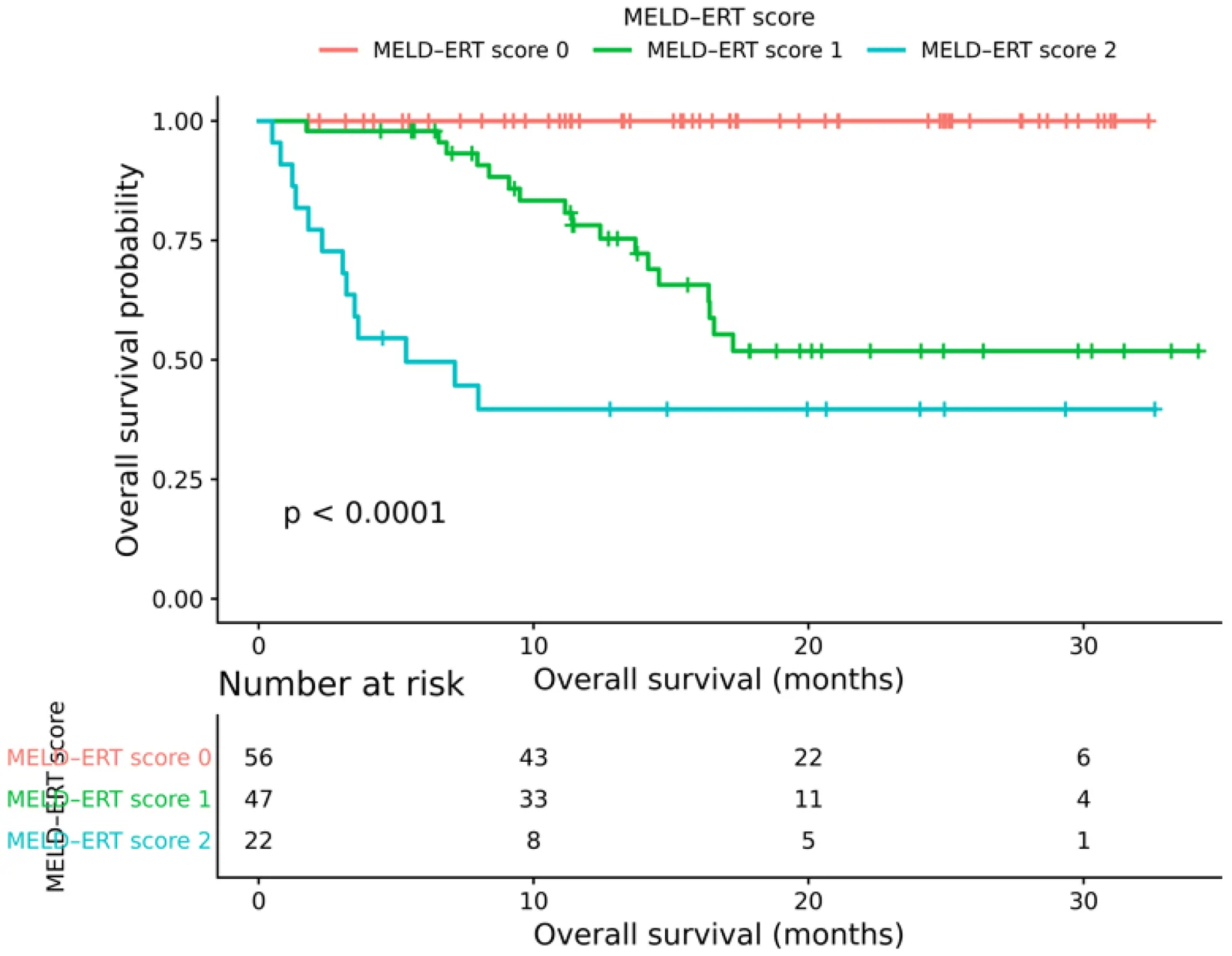
Kaplan–Meier analysis of overall survival stratified by the MELD–ERT score.

Kaplan–Meier survival analysis demonstrated significant differences in OS according to the MELD–ERT score (log-rank *p* < 0.001). OS progressively decreased with increasing MELD–ERT score. Patients with a MELD–ERT score of 2 had the poorest OS, whereas no deaths occurred among patients with a MELD–ERT score of 0 during follow-up (Figure 2).

### 3.6. Cox Proportional Hazards Regression Analysis

Univariable Cox regression analysis showed that patients with a high MELD–ERT score (score 2) had a significantly increased risk of death compared with those with scores of 0–1 (HR 5.70, 95% CI 2.76–11.79, *p* < 0.001). After adjustment for recipient age and sex, a high MELD–ERT score was associated with poorer OS (adjusted HR 5.75, 95% CI 2.77–11.94, *p* < 0.001), whereas age and sex were not significantly associated with survival (Table 6). However, the proportional hazards assumption was not satisfied for the MELD–ERT risk group; therefore, the Cox HR should be interpreted cautiously and was not considered the primary summary measure of the survival difference. Accordingly, RMST was evaluated over a 24-month time horizon. The estimated 24-month RMST was 21.2 months in the low-risk group and 11.5 months in the high-risk group. Thus, over 24 months, the high-risk group experienced an average of 9.7 fewer months of survival than the low-risk group (95% CI, 5.4–14.3 months; *p* < 0.001).

## 4. Discussion

In the present study, we developed a simple composite MELD–ERT score integrating pretransplant disease severity with intraoperative ERT burden for prognostic stratification after LT. Several important findings emerged. First, both MELD score and intraoperative ERT were independently associated with all-cause mortality. Second, mortality increased progressively across the MELD–ERT categories, from 0% in patients with a score of 0 to 36.2% in those with a score of 1 and 59.1% in those with a score of 2. Third, the MELD–ERT score demonstrated good discrimination for mortality, with an apparent AUC of 0.836 and an optimism-corrected AUC of 0.818 after bootstrap internal validation. Finally, increasing MELD–ERT scores were associated with progressively poorer overall survival. These findings suggest that integrating a measure of pretransplant disease severity with intraoperative transfusion burden may provide a simple approach to post-transplant risk stratification.

The relationship between pretransplant MELD score and post-transplant outcomes has been extensively investigated, although its prognostic performance after transplantation has been less consistent than its established role in predicting wait-list mortality. In a large cohort of 1472 liver transplant recipients, Habib et al. demonstrated that a pretransplant MELD score >25 was independently associated with poorer patient and graft survival, with the greatest effect observed during the first year after transplantation [7]. Similarly, studies focusing on recipients with very high MELD scores have demonstrated an association between increasing disease severity and early postoperative mortality [8]. Nevertheless, MELD alone provides an incomplete representation of post-transplant risk. Its ability to predict postoperative outcomes is inherently limited because it primarily reflects the recipient’s physiological condition before transplantation and does not incorporate events occurring during or immediately after the procedure [9,10].

This limitation has encouraged investigation of perioperative variables that may complement pretransplant disease severity. Intraoperative transfusion is particularly relevant in LT because substantial blood loss may occur as a consequence of portal hypertension, coagulopathy, collateral circulation, surgical complexity, and hemodynamic instability. Previous studies have consistently demonstrated an association between greater ERT requirements and adverse outcomes after LT. De Boer et al. reported that intraoperative red blood cell transfusion was associated with impaired survival after LT [11]. Similarly, perioperative ERT exposure has been associated with poorer long-term recipient survival [12]. More contemporary evidence and systematic assessments of patient blood management have continued to emphasize the association between transfusion burden and adverse outcomes following LT [13,14].

Our findings are consistent with this body of evidence. In the present cohort, each additional unit of intraoperative ERT was associated with increased odds of mortality, and this association remained significant after adjustment for MELD. This finding supports the concept that MELD and ERT capture different, but potentially complementary, dimensions of transplant risk. MELD quantifies preoperative disease severity, whereas ERT provides information arising during the transplantation procedure. However, the formal interaction analysis did not demonstrate a significant multiplicative interaction between MELD and ERT. Therefore, the rationale for the MELD–ERT score should be interpreted as the simple additive integration of two complementary prognostic dimensions rather than evidence of statistical synergy between its components. The transfusion requirement should not necessarily be interpreted as a direct causal determinant of mortality; rather, it may represent an integrated marker of surgical complexity, bleeding burden, hemodynamic disturbance, coagulopathy, and overall intraoperative physiological stress. ERT was moderately correlated with EBL. However, there was no evidence of substantial multicollinearity, and the association between ERT and mortality persisted after adjustment for EBL, suggesting that the prognostic information captured by ERT was not explained by measured blood loss alone. In the present cohort, preoperative hemoglobin, platelet count, INR, and fibrinogen were not significantly associated with intraoperative ERT requirement, and these parameters were comparable between the MELD–ERT risk groups. These findings reduce, but do not eliminate, the possibility that the prognostic association of ERT was primarily driven by baseline anemia or preoperative hematologic and coagulation abnormalities. Nevertheless, residual confounding from unmeasured intraoperative and surgical factors, including operative complexity, portal hypertension, previous abdominal surgery, hemodynamic instability, and operative duration, cannot be excluded. Studies examining bleeding and transfusion patterns in LT have similarly demonstrated close relationships among disease severity, intraoperative blood loss, transfusion requirements, and postoperative outcomes [15].

A particularly notable finding was the stepwise increase in mortality across the MELD–ERT categories. No deaths occurred among patients with neither adverse component, whereas mortality increased substantially when either one or both adverse components were present. The highest-risk group, defined by the simultaneous presence of MELD ≥ 22 and ERT ≥ 4 units, had a mortality rate of 59.1%. This gradient supports the biological and clinical rationale for combining preoperative and intraoperative information. The score was deliberately constructed as a simple unweighted additive model rather than a complex regression-derived prediction equation. Although more complex models may achieve greater statistical flexibility, parsimonious scores based on routinely available variables may be easier to interpret and potentially more practical for bedside risk stratification.

The MELD–ERT score demonstrated good discrimination for all-cause mortality, with an AUC of 0.836. However, the discrimination of MELD–ERT was not statistically superior to MELD alone. This finding is important when interpreting the potential contribution of the composite score. The principal value of MELD–ERT should therefore not be viewed as replacing MELD or demonstrating unequivocal statistical superiority over it. Rather, the score incorporates intraoperative information into an established measure of pretransplant disease severity and provides clinically intuitive risk categories. Additional model-performance analyses further support a cautious interpretation of the incremental value of MELD–ERT. Although the score showed good calibration within the development cohort, decision-curve analysis did not demonstrate a consistent incremental net benefit over MELD alone across clinically relevant threshold probabilities. Furthermore, retaining MELD and ERT as continuous variables resulted in discrimination similar to that of the simplified 0–2 score, suggesting that simplification did not substantially compromise discriminative performance. Time-dependent ROC analyses provided additional information on discrimination across follow-up. The MELD–ERT score maintained good discrimination at 3, 6, 12, and 24 months, with time-dependent AUCs ranging from 0.812 to 0.930. Notably, however, MELD–ERT did not consistently outperform MELD alone at all time points, reinforcing the interpretation that the composite score provides complementary perioperative risk stratification rather than unequivocal incremental discrimination over MELD. Previous studies evaluating postoperative MELD-based approaches and other perioperative extensions of MELD have similarly suggested that incorporation of information obtained during or after transplantation may improve characterization of postoperative risk [9,16].

The imbalance in donor type between the MELD–ERT risk groups represents another potential source of confounding. Importantly, however, the association between MELD–ERT and mortality persisted in a sensitivity analysis restricted to LDLT recipients, with a stepwise increase in mortality across score categories. This finding suggests that the observed risk gradient was not solely attributable to differences in donor type. Nevertheless, living- and deceased-donor transplantation differ in graft characteristics, ischemic times, operative complexity, and transfusion requirements. Furthermore, transfusion practices are institution-dependent; therefore, the ERT threshold of ≥4 units derived in this cohort may not be directly transferable to centers with different donor distributions or transfusion protocols.

Because the score was developed and evaluated within the same cohort, internal validation was particularly important. Bootstrap validation using 5000 resamples resulted in an optimism-corrected AUC of 0.818 compared with the apparent AUC of 0.836. The relatively small reduction in discrimination suggests limited apparent optimism and supports the internal stability of the score. Nevertheless, internal validation cannot establish transportability. Differences among transplant centers in recipient selection, donor characteristics, surgical technique, transfusion thresholds, coagulation management, and perioperative care may influence both ERT requirements and their association with outcome. Accordingly, external validation in independent cohorts remains necessary before clinical implementation can be considered.

The survival analyses provided further evidence of risk stratification by the MELD–ERT score. Kaplan–Meier curves demonstrated progressively poorer OS with increasing scores, and patients in the high-risk group had substantially worse survival than those with scores of 0–1. Importantly, the proportional hazards assumption was not satisfied for the MELD–ERT risk classification. A similar phenomenon has previously been described for ERT after LT. In a cohort of 543 recipients, Real et al. identified ERT as an independent predictor of survival but also demonstrated violation of the proportional hazards assumption. Subsequent time-varying analysis showed that the adverse association of transfusion with mortality was strongest during the early postoperative period and diminished over time [6]. This finding provides a plausible context for the non-proportional hazards observed in our cohort and suggests that the prognostic effect captured by MELD–ERT may be particularly relevant during the early post-transplant period.

For this reason, RMST was used to provide a more appropriate summary of the survival difference over a fixed 24-month time horizon. Over a 24-month time horizon, the high-risk group experienced a 9.7-month shorter RMST than the low-risk group. Unlike a conventional Cox hazard ratio, RMST does not require the proportional hazards assumption and provides an absolute measure of survival experience over a predefined period. The concordance between Kaplan–Meier separation and the RMST analysis therefore reinforces the finding that the MELD–ERT classification identifies recipients with clinically meaningful differences in survival. The time-dependent nature of transfusion-associated risk reported previously also emphasizes the importance of considering perioperative factors within the temporal context of post-transplant mortality [6].

The potential clinical utility of MELD–ERT lies in its simplicity. Because ERT becomes available only during the transplant procedure, MELD–ERT should not be considered a preoperative prognostic score; rather, it represents a perioperative risk-stratification framework that can be calculated at the completion of transplantation. At the clinically relevant transition from the operating room to postoperative intensive care, the score may provide a standardized summary of pretransplant disease severity and intraoperative transfusion burden and may facilitate postoperative risk communication and identification of patients who warrant closer surveillance. However, the present study did not demonstrate a consistent incremental clinical net benefit over MELD alone, and no specific management intervention was evaluated according to MELD–ERT category. Therefore, the score should not currently be used to mandate specific ICU monitoring or therapeutic decisions, and its clinical utility requires prospective evaluation. Previous studies have also demonstrated an association between greater intraoperative transfusion requirements and poorer post-transplant survival [17]. At the same time, contemporary patient blood management strategies emphasize minimizing avoidable transfusion while correcting clinically significant coagulopathy and maintaining adequate oxygen delivery [14,18]. The present findings should therefore not be interpreted as establishing ERT itself as a modifiable causal target based on this observational dataset, but rather as supporting its value as a readily measurable marker of perioperative risk.

Several limitations should be acknowledged. First, this was a retrospective, single-center study and is therefore susceptible to selection bias and residual confounding. Second, the sample size was relatively modest, particularly in the high-risk group, and the number of deaths limited the complexity of multivariable modeling. In addition, logistic regression analyses of all-cause mortality did not account for variation in follow-up duration and should therefore be interpreted as supportive, whereas time-to-event analyses were used to characterize survival outcomes. Third, intraoperative ERT requirement may be influenced by surgical technique, baseline coagulation status, transfusion thresholds, hemodynamic management, and institutional practice, potentially limiting the generalizability of the identified ERT threshold. Additionally, donor type was unevenly distributed across MELD–ERT risk groups; however, the association between ERT and mortality remained significant after additional adjustment for donor type in sensitivity analysis. Fourth, although bootstrap resampling provided internal validation and demonstrated limited optimism, the MELD–ERT score has not yet undergone external validation. External validation in larger, independent, multicenter cohorts, including centers from different countries and healthcare settings, is therefore warranted to assess the transportability of the score. Given the evolving nature of perioperative management and predictive modeling, future studies should also evaluate whether recalibration or refinement of the MELD–ERT score is required over time. Furthermore, no deaths occurred in the score-0 group, which may have accentuated the observed risk gradient and therefore warrants cautious interpretation. The MELD and ERT cut-offs were data-driven and derived using ROC analysis within the same cohort in which score performance was evaluated. Although bootstrap internal validation was used to quantify optimism, some degree of overfitting cannot be excluded. Dichotomization of continuous variables may also result in loss of prognostic information; however, a model retaining MELD and ERT as continuous variables showed discrimination similar to that of the simplified 0–2 score. These findings require confirmation in an independent external cohort using the same predefined thresholds.

## 5. Conclusions

The MELD–ERT score integrates pretransplant disease severity and intraoperative ERT burden into a simple prognostic classification after LT. Increasing scores were associated with progressively higher mortality and poorer OS. The score demonstrated good discrimination after bootstrap internal validation, although it did not significantly outperform MELD alone. These findings support MELD–ERT as a clinically interpretable framework incorporating both preoperative and intraoperative information. External validation in larger, multicenter cohorts is warranted.

## Figures and Tables

**Table 1:** Baseline recipient, donor, intraoperative, and outcome characteristics according to MELD–ERT risk groups.

Variable	Low MELD–ERT, Score 0–1 (*n* = 103)	High MELD–ERT, Score 2 (*n* = 22)	*p* Value
Recipient characteristics			
Age, years	56 (48.5–63)	52.5 (45.5–62.8)	0.650
Female sex, *n* (%)	33 (32.0)	9 (40.9)	0.424
BMI, kg/m^2^	25.6 (23.2–28.2)	24.4 (22.5–27.3)	0.161
Body surface area, m^2^	1.90 (1.75–2.00)	1.80 (1.70–1.90)	0.216
Diabetes mellitus, *n* (%)	27 (26.2)	6 (27.3)	0.919
MELD score	18 (16–23.5)	26 (25–30)	<0.001
Preoperative hemoglobin, g/dL	9.8 (8.8–11.0)	10.0 (9.3–10.4)	0.897
Platelet count, ×10^9^/L	88 (64–120)	81 (56–117)	0.685
INR	1.53 (1.24–1.72)	1.57 (1.32–1.82)	0.408
Fibrinogen, mg/Dl	167 (120–203)	168 (116–218)	0.894
Etiology, *n* (%)			0.831
NASH	32 (31.1)	7 (31.8)	
Alcohol-related liver disease	22 (21.4)	6 (27.3)	
Autoimmune liver disease	12 (11.7)	3 (13.6)	
HBV	17 (16.5)	4 (18.2)	
Other	20 (19.4)	2 (9.1)	
Re-transplantation, *n* (%)	2 (1.9)	1 (4.5)	0.443
Donor characteristics			
Living donor transplantation, *n* (%)	62 (60.2)	21 (95.5)	0.001
Donor age, years	42 (30.5–50.5)	33 (26.2–36)	0.001
Female donor, *n* (%)	34 (33.0)	7 (31.8)	0.914
Donor BMI, kg/m^2^	25.0 (24.2–27.0)	24.2 (23.6–26.8)	0.366
Recipient-to-donor BSA ratio	1.00 (0.90–1.10)	1.00 (0.90–1.00)	0.051
Intraoperative characteristics			
Warm ischemia time, min	4 (3–5)	4 (3–6)	0.230
Cold ischemia time, min	74 (63–380)	66.5 (48.2–75.2)	0.007
Estimated blood loss, mL	1200 (700–1900)	3000 (2125–3875)	<0.001
Cell saver returned blood, mL	598 (0–1331)	0 (0–2219.8)	0.819
Erythrocyte transfusion, units	1 (0–2)	5 (4–7.5)	<0.001
Outcomes			
Overall mortality, *n* (%)	17 (16.5)	13 (59.1)	<0.001

**Table 2:** Logistic Regression Analysis for Overall Mortality.

Variable	Univariable OR (95% CI)	*p* Value	Multivariable OR (95% CI)	*p* Value
Age (per year)	1.00 (0.97–1.04)	0.823	–	–
Female sex	1.74 (0.75–4.05)	0.198	–	–
Body mass index (per kg/m^2^)	0.92 (0.83–1.01)	0.077	–	–
Diabetes mellitus	1.57 (0.64–3.82)	0.325	–	–
Living donor transplantation	2.44 (0.91–6.54)	0.076	–	–
Donor age (per year)	0.97 (0.94–1.00)	0.067	–	–
Estimated blood loss (per 100 mL)	1.02 (0.99–1.05)	0.181	–	–
MELD score (per point)	1.19 (1.10–1.28)	<0.001	1.18 (1.10–1.28)	<0.001
Erythrocyte transfusion (per unit)	1.26 (1.07–1.49)	0.006	1.22 (1.02–1.45)	0.029

**Table 3:** MELD–ERT Sensitivity Analysis in Living Donor Liver Transplantation.

MELD–ERT Score	Patients, *n* (%)	Deaths, *n* (%)
0	32 (38.6)	0 (0.0)
1	30 (36.1)	12 (40.0)
2	21 (25.3)	12 (57.1)

**Table 4:** Prognostic Performance of the MELD–ERT Score.

MELD–ERT Score	Definition	Patients, *n* (%)	Deaths, *n* (%)
0	MELD < 22 and ERT < 4 units	56 (44.8)	0 (0.0)
1	Either MELD ≥ 22 or ERT ≥ 4 units	47 (37.6)	17 (36.2)
1A	MELD ≥ 22 and ERT < 4 units	37 (78.7) *	15 (40.5)
1B	MELD < 22 and ERT ≥ 4 units	10 (21.3) *	2 (20.0)
2	MELD ≥ 22 and ERT ≥ 4 units	22 (17.6)	13 (59.1)

* Percentages for score 1A and score 1B were calculated within the score-1 subgroup (*n* = 47); percentages for scores 0, 1, and 2 were calculated using the overall cohort (*n* = 125). Score 1A and score 1B represent exploratory subdivisions of score 1 and are not additional levels of the MELD–ERT score.

**Table 5:** Time-dependent discrimination of MELD, ERT, and MELD–ERT for post-transplant mortality.

Time Point	MELD AUC (95% CI)	ERT AUC (95% CI)	MELD–ERT AUC (95% CI)
3 months	0.857 (0.766–0.934)	0.841 (0.714–0.933)	0.893 (0.793–0.953)
6 months	0.819 (0.736–0.892)	0.875 (0.799–0.936)	0.930 (0.869–0.973)
12 months	0.789 (0.695–0.878)	0.722 (0.598–0.839)	0.837 (0.761–0.905)
24 months	0.829 (0.695–0.933)	0.537 (0.385–0.698)	0.812 (0.702–0.903)

**Table 6:** Cox Regression Analysis for Overall Survival.

Variable	Univariable HR (95% CI)	*p* Value	Multivariable HR (95% CI)	*p* Value
Age, per year	1.01 (0.98–1.03)	0.735	1.01 (0.98–1.04)	0.441
Female sex	0.64 (0.31–1.31)	0.219	0.64 (0.31–1.34)	0.239
High MELD–ERT score, 2 vs. 0–1	5.70 (2.76–11.79)	<0.001	5.75 (2.77–11.94)	<0.001

## Data Availability

The data supporting the findings of this study are available from the corresponding author upon reasonable request. The data are not publicly available due to patient privacy and ethical restrictions.
